# Editorial: Exploring the Role and Function of the Microbiota in Terrestrial Anaerobic Environments and Their Potential Biotechnological Application

**DOI:** 10.3389/fmicb.2021.722268

**Published:** 2021-07-27

**Authors:** Andreas Otto Wagner, Maria Westerholm, Blaz Stres, Jan Kopečný

**Affiliations:** ^1^Department of Microbiology, Universität Innsbruck, Innsbruck, Austria; ^2^Department of Molecular Sciences, BioCentre, Swedish University of Agricultural Sciences, Uppsala, Sweden; ^3^Department of Animal Science, Biotechnical Faculty, University of Ljubljana, Ljubljana, Slovenia; ^4^Institute of Animal Physiology and Genetics, Academy of Sciences of the Czech Republic (ASCR), Prague, Czechia

**Keywords:** anaerobe, biotechnology, application, microbial community, microbial interaction

Microorganisms play a decisive role in the carbon cycle as decomposers of a wide range of organic substances–regardless of microbial, plant, or animal origin–and are capable of using them as energy or carbon sources. Degradation processes are found under aerobic, microaerophilic as well as anoxic conditions, whereby aerobic organisms yield much more energy (ATP) compared with anaerobic ones. While the aerobic metabolism relies on the presence of oxygen as a terminal electron acceptor resulting in H_2_O and CO_2_ as degradation products, in anaerobic environments energy can be gained by using electron acceptors such as sulfate, nitrite or CO_2_ or via fermentation. Degradation and subsequent processing of organic matter is often cascade-like and consortium driven. The execution of degradation steps is facilitated by particular microbial species or by a particular succession contributing to or ensuring a smooth functioning of the overall process. Anaerobes can be found in many natural habitats including sea, lakes, soils, and sediments, in the digestive tract of animals, but also in waste water treatment plants, and biogas reactors next to pharmaceutical and food production operations, where they play an important role in the stabilization of the overall processes.

The present Research Topic “Exploring the Role and Function of the Microbiota in Terrestrial Anaerobic Environments and their Potential Biotechnological Application” comprises 7 original articles and reviews on a range of microbial services in various habitats contributed by 34 authors.

Black shank is a plant disease on tobacco plants that is caused by the oomycete pathogen *Phytophthora nicotianae*. In the study by Liu et al., the efficiency of suppressive microflora cultured from soil and roots on the growth of the *P. nicotianae* was investigated for controlling black shank. The authors concluded that microfloral communities from plant roots might be more effective in controlling black shank than those from soil, and suggested that microorganisms from the roots should be isolated and investigated for potential candidates for disease control.

In their review Du et al. summarized the recent progress on Clostridium co-culture systems, including advantages, composition, products, and interaction mechanisms, showed biochemical regulation and genetic modifications that are used to improve such culture systems, and pointed out future prospects.

Nagler et al. proposed an approach for the quantification of sequentially extracted extracellular (exDNA) and intracellular DNA (iDNA) that revealed information about cell lysis and activity of methanogenic archaea within a biogas-producing microbial community. In the study their fine-tuned DNA approach coupled with the interpretation of the ratio between free exDNA and iDNA considerably improved microbial activity tracking compared to the classical extraction approaches and allowed to draw conclusions on the inactive part of a methanogenic population.

In the study by Umair et al. the responses of karst microbial communities to variations in soil moisture (wet and dry season) were documented via water addition experiments. During the dry season, fungal and bacterial communities were significantly distinct from those during the wet season. Water addition did not alter the composition of bacterial or fungal communities during the dry season while during the wet season, bacterial communities were impacted. By the addition of water during the wet season changes in soil K and Na contents were observed influencing bacterial communities, which seemed to be more sensitive to variations in soil moisture in contrast to fungal ones.

The mechanisms of *Lactobacillus plantarum* involved in the improvement of fermentation quality of naturally ensiled alfalfa under poor conditions were studied by Yang et al. High-moisture wilted alfalfa was ensiled with or without inoculants of two *Lactobacillus plantarum* additives, the ensiling process monitored and the bacterial community composition analyzed. The inoculants significantly promoted lactic acid accumulation and the abundance of *Lactobacillus plantarum*, whereas the growth of Clostridia was inhibited and ammoniacal nitrogen concentration reduced.

Mutschlechner et al. explored and compared the potential to use soil-derived microbial communities as inoculum for anaerobic digestion processes. The highest CH_4_ production was observed using unsterile soil combined with sterile sludge. The higher methane yield coincided with both, a higher relative abundance of methanogens and predicted genes involved in CH_4_ metabolism.

Yang et al. investigated the bacterial composition in alfalfa silage with and without inoculation by *Lactobacillus plantarum* after 10 and 60 days using absolute quantification 16S-sequencing (AQS) and relative quantification 16S-sequencing (RQS). AQS indicated higher bacterial richness indices and closer correlations of these indices with fermentation properties than RQS. AQS could be used effectively to illustrate the dynamics of bacterial communities during the ensiling process.

In summary, the articles included in this Research Topic demonstrate the importance of microorganisms in various microaerobic and/or anaerobic habitats. The broad range of presented articles deepen our knowledge on role and function of anaerobes in these habitats, either in pure-, co-, or mixed cultures, and provide exciting insights into research progress also including methodical issues. The outcome of such studies emphasizes the enormous potential of microbial services in decomposition processes but also for biotechnological applications.

## Author Contributions

AW, MW, BS, and JK wrote the manuscript. All authors read and approved the final manuscript.

## Conflict of Interest

The authors declare that the research was conducted in the absence of any commercial or financial relationships that could be construed as a potential conflict of interest.

## Publisher's Note

All claims expressed in this article are solely those of the authors and do not necessarily represent those of their affiliated organizations, or those of the publisher, the editors and the reviewers. Any product that may be evaluated in this article, or claim that may be made by its manufacturer, is not guaranteed or endorsed by the publisher.

